# Overexpressing 3-Hydroxy-3-Methylglutaryl Coenzyme A Reductase (HMGR) in the Lactococcal Mevalonate Pathway for Heterologous Plant Sesquiterpene Production

**DOI:** 10.1371/journal.pone.0052444

**Published:** 2012-12-26

**Authors:** Adelene Ai-Lian Song, Janna Ong Abdullah, Mohd. Puad Abdullah, Norazizah Shafee, Roohaida Othman, Ee-Fun Tan, Normah Mohd. Noor, Abdul Rahim Raha

**Affiliations:** 1 Department of Cell and Molecular Biology, Faculty of Biotechnology and Biomolecular Sciences, Universiti Putra Malaysia, Serdang, Selangor Darul Ehsan, Malaysia; 2 Department of Microbiology, Faculty of Biotechnology and Biomolecular Sciences, Universiti Putra Malaysia, Serdang, Selangor Darul Ehsan, Malaysia; 3 Institute of Bioscience, Universiti Putra Malaysia, Serdang, Selangor Darul Ehsan, Malaysia; 4 Institute of Systems Biology, Universiti Kebangsaan Malaysia, Bangi, Selangor Darul Ehsan, Malaysia; 5 School of Biosciences and Biotechnology, Faculty of Science and Technology, Universiti Kebangsaan Malaysia, Bangi, Selangor Darul Ehsan, Malaysia; Belgian Nuclear Research Centre SCK/CEN, Belgium

## Abstract

Isoprenoids are a large and diverse group of metabolites with interesting properties such as flavour, fragrance and therapeutic properties. They are produced via two pathways, the mevalonate pathway or the 2-C-methyl-D-erythritol-4-phosphate (MEP) pathway. While plants are the richest source of isoprenoids, they are not the most efficient producers. *Escherichia coli* and yeasts have been extensively studied as heterologous hosts for plant isoprenoids production. In the current study, we describe the usage of the food grade *Lactococcus lactis* as a potential heterologous host for the production of sesquiterpenes from a local herbaceous Malaysian plant, *Persicaria minor* (synonym *Polygonum minus*). A sesquiterpene synthase gene from *P. minor* was successfully cloned and expressed in *L. lactis*. The expressed protein was identified to be a β-sesquiphellandrene synthase as it was demonstrated to be functional in producing β-sesquiphellandrene at 85.4% of the total sesquiterpenes produced based on *in vitro* enzymatic assays. The recombinant *L. lactis* strain developed in this study was also capable of producing β-sesquiphellandrene *in vivo* without exogenous substrates supplementation. In addition, overexpression of the strain’s endogenous 3-hydroxy-3-methylglutaryl coenzyme-A reductase (HMGR), an established rate-limiting enzyme in the eukaryotic mevalonate pathway, increased the production level of β-sesquiphellandrene by 1.25–1.60 fold. The highest amount achieved was 33 nM at 2 h post-induction.

## Introduction

*Lactococcus lactis* is a food grade lactic acid bacterium which has been used as a starter culture in food fermentation for centuries. However, over the last decade, *L. lactis* has found other new and exciting applications as it has been developed into cell factories for the production of bioactive compounds such as enzymes, peptides and vaccine antigens [1], [2]. Recently, Hernandez *et al*. [3] reported on the first attempt to utilize *L. lactis* for the production of the isoprenoids linalool and nerolidol from strawberry (*Fragaria×ananassa*) by heterologous expression of the *FaNES* gene.

Isoprenoids are a large and diverse group of naturally occurring metabolites found in the primary metabolism of all living organisms. However, some organisms especially plants also produce isoprenoids as secondary metabolites, and these isoprenoids have interesting properties such as flavour, fragrance and toxicity [4]. Some of the most valuable isoprenoids known so far include the diterpene, taxadiene, which is the precursor for paclitaxel, a potent anti-cancer drug as well as amorphadiene, the sesquiterpene precursor for the anti-malarial drug artemisinin [5]. Isoprenoids may be synthesized via two pathways, either the mevalonate pathway or the non-mevalonate pathway, also known as the 2-C-methyl-D-erythritol-4-phosphate (MEP) pathway [6]. All organisms only have either one of the pathways except plants which possess both pathways, although they are localized in different organelles of the cell. The mevalonate pathway is usually associated with eukaryotes while the MEP pathway is more commonly found in prokaryotes. However, some Gram-positive bacteria such as *Staphylococcus aureus*, *Lactobacillus plantarum* and *L. lactis* have adopted the mevalonate pathway through evolution for isoprenoid biosynthesis [7], [8].

*E. coli* and yeasts have been extensively studied and engineered as heterologous hosts for the production of plant isoprenoids. These include many different metabolic engineering strategies employed to increase the production of the heterologous isoprenoids in the host cells [9]. However, *L. lactis* is also a potential heterologous host for isoprenoid production with certain advantages such as its GRAS (Generally Regarded as Safe) status and the absence of inclusion bodies [3], [10]. Apart from that, *L. lactis* offers a food-grade alternative to the other more common and well-studied hosts for heterologous isoprenoid production such as *E. coli* and *S. cerevisiae*. Whilst *E. coli* uses the MEP pathway and yeast uses the mevalonate pathway, respectively, for the production of isoprenoids, the study of *L. lactis* as a prokaryotic host which uses the mevalonate pathway for isoprenoid production is appealing.

Previously, we reported the cloning and expression of a sesquiterpene synthase from orchid (*Vanda* Mimi Palmer) in *L. lactis* [10]. Here we describe the cloning and expression of another sesquiterpene synthase, isolated from *Persicaria minor* (synonym *Polygonum minus*), a local Malaysian herbaceous fragrant plant with biological activities including anti-oxidant [11] and anti-ulcer [12] properties. The recombinant *L. lactis* developed was able to produce *P. minor* sesquiterpene *in vivo* thereby making it suitable as a microbial cell factory for *P. minor* sesquiterpene production. We also attempted to metabolically engineer the mevalonate pathway of *L. lactis* by overexpressing the endogenous 3-hydroxy-3-methylglutaryl coenzyme-A reductase (HMGR) enzyme, an established rate-limiting enzyme in the eukaryotic mevalonate pathway [13]. This is the first reported attempt to metabolically engineer the endogenous host’s prokaryotic mevalonate pathway for an increased isoprenoid production.

## Materials and Methods

### Bacterial Strains and Culture Conditions

*L. lactis* NZ9000 strain, a derivative of the nisin-negative *L. lactis* MG1363 which has the n*is*R and *nis*K genes from the nisin gene cluster inserted into its chromosome was used as the host in this study [14]. The *nis*R and *nis*K genes enable nisin induction of the PnisA promoter on the pNZ8048 plasmid which was used for all *L. lactis* cloning purposes in this study. All *L. lactis* strains were cultured in M17 broth or agar [15] supplemented with 0.5% (w/v) glucose (GM17) and 7.5 µg/mL chloramphenicol whenever necessary. *L. lactis* was typically grown at 30°C as a stand culture. When screening for *L. lactis* transformants, M17 agar supplemented with 0.5% (w/v) glucose, 0.5 M sucrose and 7.5 µg/mL chloramphenicol was used.

### Gene Amplification and Plasmids Construction

The *P. minor* sesquiterpene synthase (PMSTS) [Accession no: JX025008] was amplified using the PMSTS forward and reverse primers 5′- CTCTGCAGAA*CATCACCATCACCAT CAC*ATGTATTCCATGATCATG-3′ and Rev: 5′- CTGGTACCTTATATCAGTATGGG ATCG-3′ primers respectively. The restriction enzyme (RE) sites, *Pst*I and *Kpn*I are underlined while the his-tag sequence incorporated into the forward primer is shown in italic. The *mvaA* gene was amplified using nested PCR from the genome of *L. lactis* MG1363 [Accession no: YP_001032255]. Due to the highly rich A-T sequence in the *mvaA* gene, a longer *mva*A fragment which included flanking regions were first amplified and the PCR product was susbsequently used as template for a second amplification of the actual *mvaA* gene using the forward and reverse primers 5′- CTGGTACCGGAGGCAAAAAATGATGAGAA AAAAATTTTATCAAATGTC 3′ and 5′-CTAAGCTTCTA*ATGGTGATGGTGATG GTG*TTTTCTCAAATTTTTTAGTAAATTTTGG- 3′ respectively. As with the PMSTS primers, the RE sites, *Kpn*I and *Hin*dIII, are underlined and the his-tag sequence incorporated into the reverse primer is italicized. A ribosomal binding site (RBS), highlighted in grey is also incorporated into the forward primer. The PCR reaction mixture contained 1× reaction buffer, 2 mM MgCl _2_, 0.2 mM dNTP, 5 units of Taq polymerase or High-Fidelity PCR Enzyme Mix (Fermentas, USA), 0.5 µM of forward and reverse primers and approximately 20 ng of template DNA. The reaction was run for 30 cycles using a 94°C for 1 min, 60°C for 1 min and 72°C for 2 min temperature-time profile. To construct the pNZ:*PMSTS* plasmid, the digested PMSTS gene was ligated into pNZ8048 digested with *Pst*I and *Kpn*I and then transformed into the *L. lactis* NZ9000 host via electroporation [16]. To construct the pNZ:*PMSTS*:*mvaA* plasmid, the amplified *PMSTS* and *mvaA* genes digested with their respective REs were allowed to ligate with pNZ8048 digested with *Pst*I and *Hin*dIII and then transformed into *L. lactis* NZ9000. This allowed the *mvaA* gene to be ligated downstream of the *PMSTS* gene via the *Kpn*I site in the pNZ8048 plasmid, thereby yielding the pNZ:*PMSTS*:*mvaA* plasmid.

### Expression of Recombinant Proteins

For protein expression, overnight culture of *L.lactis* NZ9000 harbouring pNZ:*PMSTS* or pNZ:*PMSTS*:*mvaA* were inoculated into fresh GM17 medium at 5% (v/v) and grown to an OD600 of 0.4. The culture was induced with 40 ng/mL nisin for 2 h which was pre-determined to be the optimum induction condition (data not shown). After induction, the cells were harvested in an assay buffer (20 mM Tris-HCl, pH 8.0, 5 mM MgCl_2_ and 2 mM dithiothreitol), modified from Chen *et al*. [17], by centrifugation at 1438×g for 10 min at 4°C. Crude extracts were prepared by subjecting the cells to sonication treatment using the Omni Ruptor 4000 (Omni International, GA, USA) set to 10% power and pulsed for 2 min per sample. Then, the samples were centrifuged at 16,000×g, for 10 min, at 4°C with the final aqueous phase collected for further purification. For recombinant protein purification, His SpinTrap (GE Healthcare, WI, USA) columns equilibrated with ten column volumes of binding buffer containing 20 mM imidazole were used according to the manufacturer’s protocol to trap recombinant protein via the his-tag fusion. Subsequently, the column was washed with another ten column volumes of binding buffer and the final elution was performed in the presence of 300 mM imidazole. After purification, the proteins were desalted using HiTrap Column (GE Healthcare, WI, USA) according to the manufacturer’s protocol. At each step, the protein was analyzed by SDS-PAGE according to Laemmli [18].

### *In vitro* Enzymatic Assays

Enzymatic assay for the recombinant sesquiterpene synthase was performed as previously described [17]. Briefly, the purified protein extract in the assay buffer was mixed with 20 µL farnesyl diphosphate substrate from a 1 µg/µL stock (Sigma Aldrich, MO, USA) in a total volume of 1 mL. The mixture was then incubated with shaking at 30°C for 3 h and the reaction was stopped with 1 mL of 4 M CaCl_ 2_. All the above reactions were performed in a 20 mL headspace vial which was then subjected to solid phase microextraction (SPME) sampling using a 100 µm polydimethylsiloxane (PDMS) coated fiber. Extraction time used was 15 min at 60°C and desorption time was 5 min. Blank tests were run in between sampling runs to ensure no carryover from the SPME fiber or the GC-MS system.

HMGR enzymatic assays were performed according to Wilding *et al*. [19] with minor modifications. His-tag purified protein from pNZ:*PMSTS*:*mvaA* was added to the assay buffer (50 mM NaCl, I mM EDTA, 25 mM KH_2_PO_4_, pH 7.5, 5 mM DTT, 0.25 mM NADPH and 0.25 mM HMG-CoA). The protein sample in assay buffer mixture was diluted to a final volume of 1 mL. Spectrophotometric assays were performed using a Hitachi U-2900 Spectrophotometer (Hitachi, Tokyo, Japan) set to read OD measurements at 340 nm over a time period of 3 min. The HMG-CoA was added last to initiate the reaction just before spectrophotometer readings were taken. Purified his-tag protein from pNZ:*PMSTS* clones were used as a negative control.

### GC-MS Analysis

Products from the enzymatic assays were analyzed using the GC-MS Turbomass Clarus 600 (Perkin Elmer, MA, USA) and the GC column used was Perkin Elmer Elite 5 MS (30 m×0.25 mm ID). The GC oven conditions were from an initial temperature of 40°C (1 min hold) to 250°C (3 min hold) with a 35°C ramping temperature increase per min. The column flow was held constant at 1 mL/min. The injection port was maintained at 250°C, the transfer line at 220°C while the MS source temperature was maintained at 200°C. The MS, operated at 70 eV was set to scan from m/z 35 to 400. Products were identified by comparing the spectra to those in the National Institute of Standards and Technology (NIST) library 2008 and the Wiley Registry of Mass Spectral Data, 8^th^ Edition. The cut-off match score for the mass spectra of the products compared to the NIST and Wiley libraries used were >800.

### *In vivo* Expression

Overnight culture of *L. lactis* NZ9000 harbouring pNZ:*PMSTS*, pNZ:*PMSTS*:*mvaA* or empty pNZ8048 (as negative control) were inoculated into fresh GM17 medium at 5% (v/v) and grown to an OD_600_ of 0.4. The cultures were split into sub-samples consisting of uninduced samples and samples induced with nisin at 40 ng/mL. During induction, 100 mL of each culture was transferred into 150 mL serum bottles, spiked with chlorohexane as internal control and capped with a crimp top aluminium cap. To measure the sesquiterpenes produced, the headspace of the serum bottle was subjected to SPME sampling at 60°C for 15 min hourly from 1–4 h post-induction. The PDMS fibre was then subjected to GC-MS analysis. Production of sesquiterpenes were measured based on peak area of GC-MS peaks, normalized against chlorohexane, the internal standard and quantified as nerolidol equivalents using nerolidol standards (Sigma, MO, USA) since standards for the sesquiterpenes produced by the recombinant clones in this study were commercially unavailable [20]. Five biological replicates were done for each experiment.

## Results and Discussion

### Plasmids Construction

**Figure 1:**
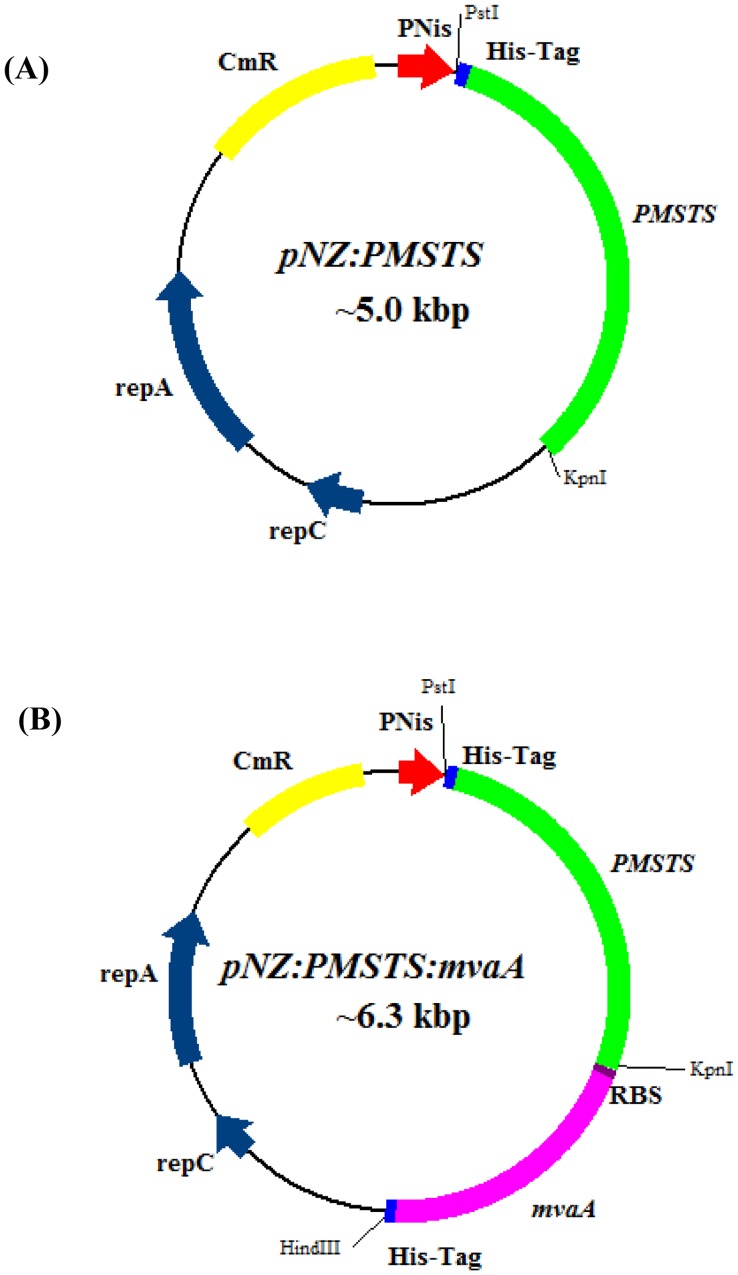
Schematic diagram of pNZ:*PMSTS* (A) and pNZ:*PMSTS*:*mvaA* (B) plasmids.

Two plasmids were constructed in this study, pNZ:*PMSTS* and pNZ:*PMSTS*:*mvaA*. The pNZ:*PMSTS* plasmid harbours the heterologous sesquiterpene synthase gene, *PMSTS* from *P. minor* incorporated into the pNZ8048 plasmid (Figure 1). Two base substitutions, 796A>G and 1077A>G, were introduced into the *PMSTS* gene during amplification resulting in an amino acid change from lysine to glutamate (K266E) for the former while the latter caused a silent mutation (alanine remains as the original amino acid). An *mvaA* gene, which codes for the 3-hydroxy-3-methylglutaryl coenzyme-A reductase (HMGR), was cloned downstream of the *PMSTS* gene to yield the recombinant plasmid pNZ:*PMSTS*:*mvaA*. This construct was used for the HMGR overexpression study. Both *PMSTS* and *mvaA* genes possess a his-tag sequence for purification purposes. For *PMSTS*, the his-tag sequence is at the N-terminus while for *mvaA*, it is located at the C-terminus. In addition, an RBS site was incorporated up-stream of the *mvaA* gene to allow translation of this second gene using the same pNis promoter.

### Production of Recombinant PMSTS and HMGR Enzymes

**Figure 2:**
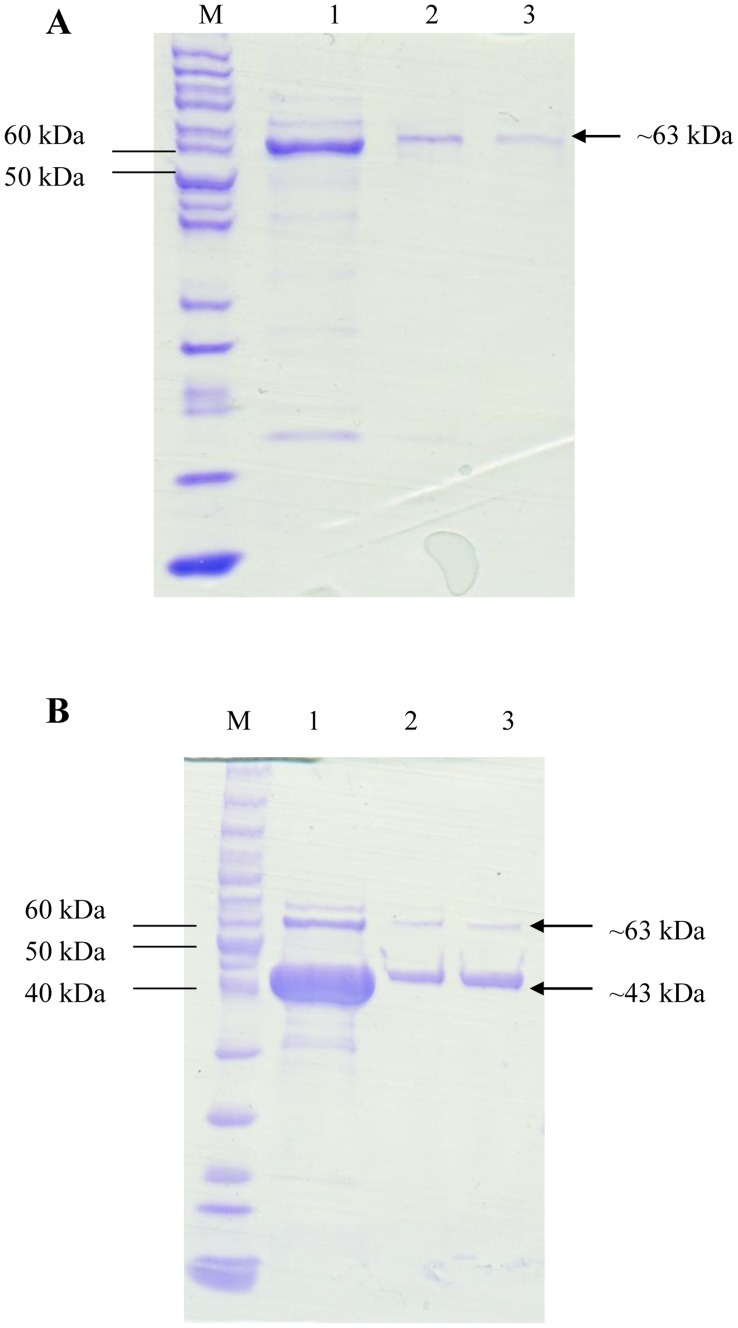
His-tag purified PMSTS and HMGR proteins (A) pNZ:*PMSTS* and (B) pNZ:*PMSTS*:*mvaA* plasmids. Lane M: PageRuler™ Prestained Plus Protein Ladder (Fermentas, Canada); Lane 1: His-tag purified protein in Elution Buffer with 300 mM imidazole; Lanes 2 and 3: Protein from Lane 1 after desalting (eluents 1 and 2 respectively).

The recombinant PMSTS from the pNZ:*PMSTS* plasmid and PMSTS together with HMGR from the pNZ:*PMSTS*:*mvaA* plasmid were analysed by SDS-PAGE after protein purification and were found to be successfully produced (Figure 2). As expected, the pNZ:*PMSTS* plasmid produced only one intense PMSTS protein band of ∼63 kDa. However, some host protein contaminations can be seen from the presence of faint bands of different sizes in Figure 2A, lane 1. These faint bands were undetected after the desalting process due to their low concentrations and only the PMSTS band remained (Figure 2A, Lanes 2 and 3). On the other hand, the pNZ:*PMSTS*:*mvaA* plasmid produced two separate intense protein bands ∼63 kDa for the PMSTS protein and ∼43 kDa for the HMGR protein (Figure 2B). The HMGR protein band is much more intense compared to the PMSTS band. This is not surprising since the HMGR protein is expressed from the *mvaA* gene which is an endogenous *L. lactis* gene while the *PMSTS* gene is a plant heterologous gene. Therefore, it was expected that the *L. lactis* host would be able to express the *mvaA* gene more efficiently since it was not limited by factors such as codon bias which would affect the PMSTS protein synthesis levels. As per the pNZ:*PMSTS* plasmid, there were also some host protein contaminations in the initial his-tag purified fraction which were subsequently not observed after the desalting step.

### Terpene Synthase Enzymatic Assay

**Figure 3:**
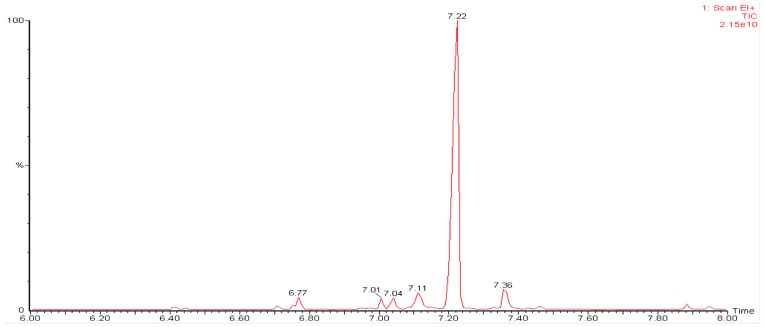
GC-MS chromatogram showing sesquiterpenes produced from purified PMSTS protein. Summary of retention times, match score and percentages of sesquiterpenes produced are shown in the Table 1.

**Figure 4:**
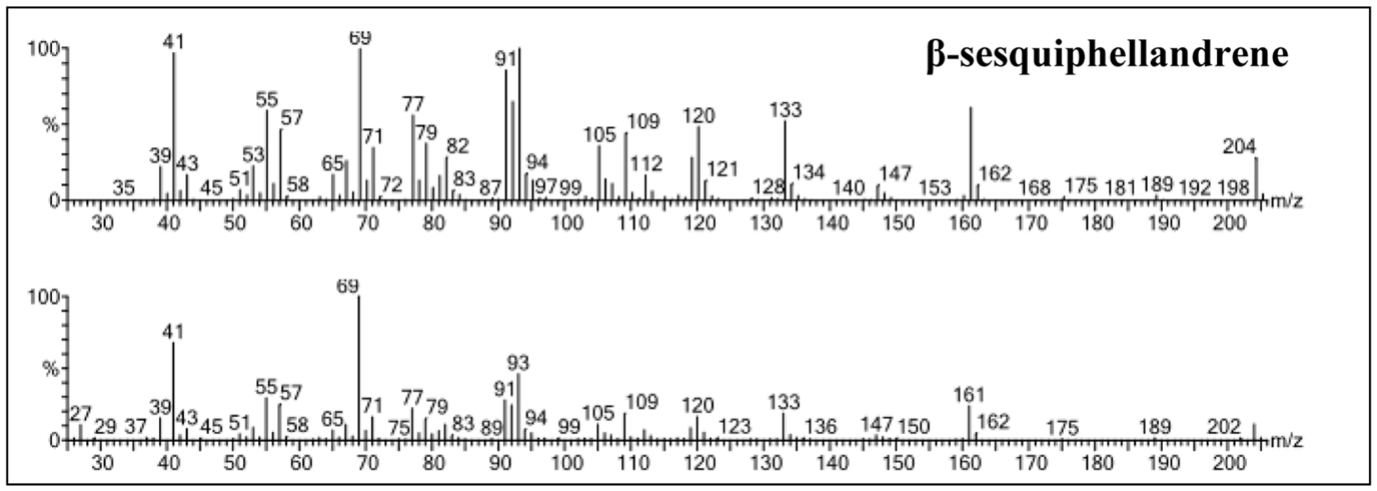
Mass spectra of the β-sesquiphellandrene. Top panel shows mass spectra of the sample compound while bottom panel shows mass spectra of the highest hit in the National Institute of Standards and Technology (NIST) library 2008 and the Wiley Registry of Mass Spectral Data, 8th Edition mass spectra library.

To assess the function of the recombinant PMSTS protein, enzymatic assays were performed using the his-tag purified and desalted PMSTS protein. The PMSTS protein was found to produce mainly β-sesquiphellandrene which consisted of 85.4% of the total sesquiterpenes produced (Figure 3) as shown by the high peak at retention time (RT) 7.22 min. Other minor products produced are shown in Table 1 while the mass spectra of the β-sesquiphellandrene peak in comparison with the highest hit from the NIST 2008 and Wiley Registry of Mass Spectral Data, 8^th^ edition library is shown in Figure 4. All peaks were identified by comparison of the mass spectra with the NIST and Wiley libraries only. However, future work to confirm and complete the chemical characterisation of the β-sesquiphellandrene peak identified in this study through NMR and X-ray diffraction studies is suggested.

**Table 1:** Sesquiterpenes produced by the recombinant PMSTS protein in *in vitro* enzymatic assay as detected by GC-MS.

Sesquiterpenes	RT (min)	Match score	Percentage (%)
α-bergamotene	6.708	946	0.8
(Z)-beta-farnesene	6.769	938	3.2
(E)-beta-farnesene	7.006	901	2.0
α-zingiberene	7.041	943	2.6
β-sesquiphellandrene	7.225	913	85.4
(E)-nerolidol	7.356	958	6.0

β-sesquiphellandrene has been previously detected from other plant terpene synthases such as ginger (*Zingiber officinale*) [21], [22] and grapevine (*Vitis vinifera*) [23]. However, in grapevine, β-sesquiphellandrene was produced as a secondary sesquiterpene by an α-zingiberene synthase, VvPNaZin. This sesquiterpene synthase produced 79.5% α-zingiberene, 17.5% β-sesquiphellandrene and 3% β-bisabolene. In fact, to our knowledge, there has not been any sesquiterpene synthase isolated or identified which produced β-sesquiphellandrene as its major product. Whether this is the genuine product of the PMSTS isolated from *P. minor*, caused by the single amino acid substitution (K266E) introduced during the cloning process or an effect of the lactococcal host expressing the recombinant protein is yet to be resolved. It should also be noted that β-sesquiphellandrene was not detected in the essential oil of the *P. minor* plant itself, although all the other minor products detected from the *in vitro* assay of PMSTS was detected in the plant [24]. This further supports the hypothesis that the mutation or host may have affected the major product produced by PMSTS although it could also be argued that the extraction or detection method used by Baharom *et al.* [24] was not sensitive towards β-sesquiphellandrene detection. As can be seen from their study, analysis using GC-MS and GCxGC-TOF MS produced significant differences in the products detected in the essential oil of *P. minor*. However, it was previously reported that a single point mutation in the active site of an aristolochene synthase was sufficient to convert it into an (E)-β-farnesene synthase instead [25]. In the current study, the K266E mutation is ten amino acids upstream of the predicted substrate binding pocket based on sequence analysis. While the C-terminal domain of terpene synthases generally contain the active site, mutational analysis of the N-terminal domain indicates that the the domain facilitates proper folding of the active site [26]. Since a substitution from lysine to glutamate changes the amino acid charge from positive to negative, the effect on protein folding may be significant and may change the catalytic activity of the enzyme.

While many essential oils which contain β-sesquiphellandrene have been shown to possess anti-microbial and anti-oxidant activities [22], [27], the effect may be caused by other components of the essential oil especially in cases where β-sesquiphellandrene is found only in minute amounts. Denyer *et al.* [21], however, showed that among α-zingiberene, β-sesquiphellandrene and β-bisabolene from the essential oils of ginger, β-sesquiphellandrene had the highest antirhinoviral activity with an IC_50_ of 0.44 µM in the plaque reduction test [21].

### HMGR Enzymatic Assay

**Figure 5:**
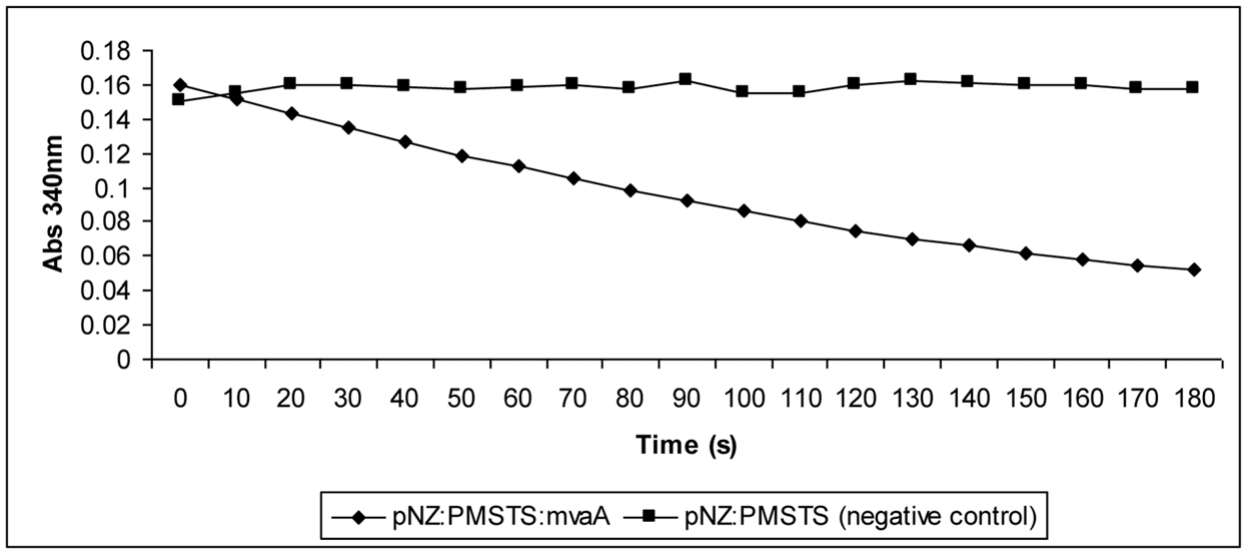
HMGR assay using his-tag purified protein expressed from pNZ:*PMSTS*:*mvaA*. Protein expressed from his-tag purified pNZ:*PMSTS* was used as the negative control.

A HMGR assay was performed to determine the functionality of the endogenous HMGR protein which was produced by overexpressing the *mvaA* gene downstream of the *PMSTS* gene in the plasmid pNZ:*PMSTS*:*mvaA*. While the HMGR protein was successfully produced as shown in Figure 2B, the functionality of the enzyme remained unknown. Therefore, the purified protein from pNZ:*PMSTS*:*mvaA* was used to perform the HMGR assay. Essentially, the purified protein represents the HMGR protein expressed from the plasmid together with the PMSTS protein without the HMGR protein expressed from the host genome. The plasmid-expressed HMGR, if functional, would be responsible for the reduction of the substrate HMG-CoA to mevalonate which is coupled to the oxidation of NADPH to NADP^+^. Figure 5 showed a decrease in absorbance at 340 nm can be observed from the oxidation of NADPH to NADP+, proving that the HMGR enzyme expressed from the plasmid was indeed functional. Negative control was also performed with his-tag purified protein from the pNZ:*PMSTS* which did not contain the HMGR protein.

### *In vivo* Production of β-sesquiphellandrene

**Figure 6:**
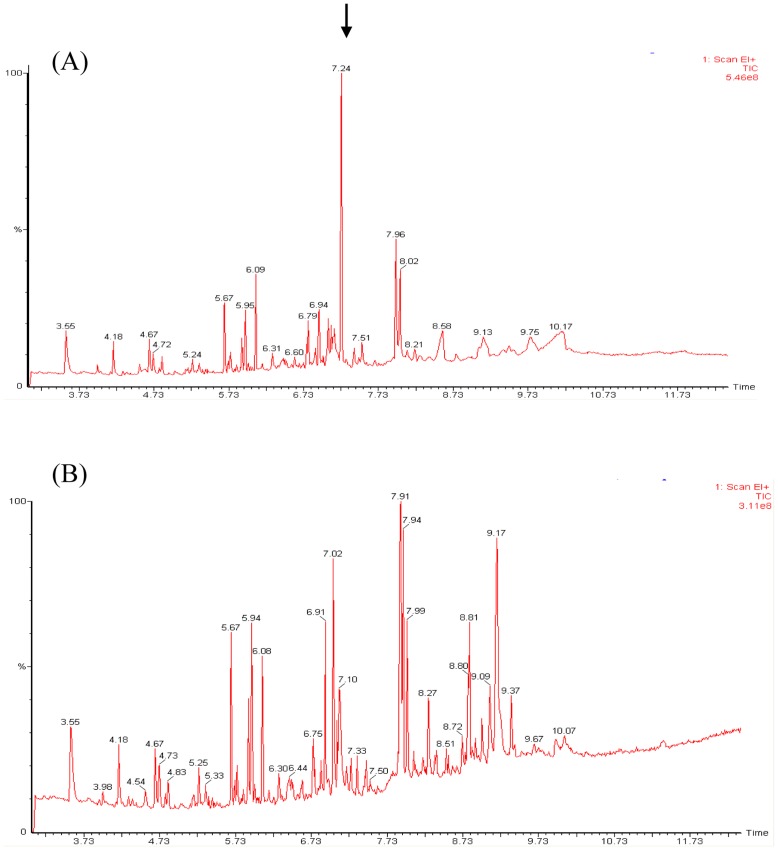
GC-MS chromatogram showing the *in vivo* production of β-sesquiphellandrene by clones harbouring the pNZ:*PMSTS* plasmid. The example shown here is for 40 ng/mL nisin induction at 2 h post-induction. B-sesquiphellandrene was detected at 7.24 min in the headspace of the induced culture (indicated by arrow) (A), which was undetected in the uninduced culture (B). Clones harbouring the pNZ:*PMSTS*:*mvaA* plasmid showed a similar GC-MS profile (data not shown).

Recombinant *L. lactis* strains harbouring the pNZ:*PMSTS* and pNZ:*PMSTS*:*mvaA* plasmids were analysed to assess its capability to produce *P. minor* sesquiterpenes *in vivo*. Upon induction with 40 ng/mL nisin, it was observed that the recombinant *L. lactis* strains were capable of producing β-sesquiphellandrene, the major product of the recombinant PMSTS protein without any exogenous FPP substrate supplementation. The β-sesquiphellandrene peak was observed at RT 7.24 min (Figure 6A) and this peak was undetected in the uninduced culture (Figure 6B). The other minor sesquiterpene products, however, were not detected from the recombinant *L. lactis* cultures probably due to their low concentrations. The clones harbouring the pNZ:*PMSTS*:*mvaA* plasmid were expected to produce more β-sesquiphellandrene compared to the clones harbouring the pNZ:*PMSTS* plasmid based on the assumption that overexpression of the HMGR enzyme encoded by the *mvaA* gene would enhance production of the downstream products. Apart from having multiple copies of the *mvaA* gene, since *mvaA* was cloned into the high copy-number plasmid pNZ8048, it was hoped that the HMGR would also be able to by-pass endogenous host regulation at least at the transcriptional level since transcription would be controlled by the plamid promoter, PNis. This metabolic engineering strategy is commonly used to increase isoprenoid production in yeasts and fungi [28], [29] ever since it was established that HMGR is the rate-limiting enzyme in the eukaryotic pathway. Considering the lack of reported work on the prokaryotic mevalonate pathway, the current study attempted to analyse the overexpression effect of HMGR on isoprenoid production in a prokaryotic system. β-sesquiphellandrene concentrations produced by clones harbouring the pNZ:*PMSTS* plasmid were compared to clones harbouring the pNZ:*PMSTS*:*mvaA* plasmid. The concentration of β-sesquiphellandrene produced was measured as nerolidol equivalents since the standard for β-sesquiphellandrene is not commercially available. Such measure was similarly practised by Pitera *et al*. [20] to quantify amorphadiene concentrations using caryophyllene equivalents.

As shown in Table 2, the clone harbouring pNZ:*PMSTS*:*mvaA* produced the highest level of 33.7 nM β-sesquiphellandrene after 1 h of induction. The overall production trend showed a gradual decline following the maximal peak production. This production pattern was in line with previous reports observing that heterologous isoprenoids production *in vivo* followed an exponential growth and ceased at the stationary phase due to the production of the FDP substrate during growth for primary metabolism [30]. For the clone harbouring pNZ:*PMSTS*, overall production was slightly lower than pNZ:*PMSTS*:*mvaA*, with the highest production being 30.0 nM sesquiphellandrene at 2 h post-induction, meeting the same amount of sesquiphellandrene production by pNZ:*PMSTS*:*mvaA* at that time. The biggest difference in production between the two clones was achieved at 1 h post-induction with pNZ:*PMSTS*:*mvaA* producing about 1.60 folds more sesquiphellandrene than pNZ:*PMSTS*. However, the overall production of pNZ:*PMSTS*:*mvaA* throughout the 4 h was only 1.25 folds more than those produced by pNZ:*PMSTS*.

**Table 2:** *In vivo* production of β-sesquiphellandrene comparing clones harbouring the pNZ:*PMSTS*:*mvaA* and the pNZ:*PMSTS* plasmids.

	Production of β-sesquiphellandrene (nM)				
Clones	1 h	2 h	3 h	4 h	Total
pNZ:*PMSTS:mvaA*	33.7	29.1	24.5	21.7	109.0
pNZ:*PMSTS*	20.9	30.0	21.3	15.0	87.2

Data shown is the mean ± standard deviation (SD) of five biological replicates.

Previous studies have also shown the ineffectiveness of the overexpression of HMGR alone to increase production of isoprenoids significantly, especially if the product of interest is further downstream of the pathway such as in the production of triterpenes [31]. Even in the production of sesquiterpenes, Jackson *et al*. [32] observed that the overexpression of a truncated HMGR along with co-expression of an epi-cedrol synthase in yeast produced significantly less epi-cedrol compared to yeast expressing the epi-cedrol synthase alone. Only with synergistic overexpression of the truncated HMGR and an *upc2-1* mutation of the yeast strain was the production of epi-cedrol increased significantly about 4-fold. Rico *et al*. [33] also found that the overexpression of HMGR alone doubled the production of the monoterpene linalool, just slightly higher than the amount observed in the current study. Regardless of the effectiveness of the overexpression of the HMGR in isoprenoid production, it has been well established that HMGR is indeed the rate-limiting enzyme in eukaryotes and studies have also shown that HMGR is the most effective gene to overexpress for prenyl alcohol production in *S. cerevisiae* in comparison with other genes of the mevalonate pathway [34]. However, studies on the prokaryotic mevalonate pathway are severely lacking with only one study done thus far on the mevalonate pathway of *S. aureus* [35]. It was suggested that unlike the eukaryotic mevalonate pathway, the rate-limiting enzyme in *S. aureus* is not HMGR but the *mvaK1-mvaD-mvaK2* operon instead which is slightly further downstream of the mevalonate pathway. Therefore, in the current study, the reason the overexpression of HMGR only yielded a slight increase of sesquiterpene production in *L. lactis* harbouring the pNZ:*PMSTS:mvaA* plasmid could be due to the fact that the HMGR in *L. lactis* is similarly not the rate-limiting enzyme and there are other metabolic bottle-necks further downstream of the mevalonate pathway.

Apart from that, while ideally it is postulated that a single optimised heterologous system can be used for overproduction of a variety of isoprenoids, we found that this may not always be true. Our previous study [10] involving the cloning and expression of a sesquiterpene synthase from orchid (*Vanda* Mimi Palmer) did not yield any plant sesquiterpenes *in vivo* although *in vitro* enzymatic studies showed that the recombinant protein was functional and the protein expression levels were three times more than the expression of PMSTS in the current study (data not shown). This shows that the regulation of the prokaryotic mevalonate pathway is a complex process which needs further studies to be understood and we are currently working on enhancing our understanding of the mevalonate pathway of *L. lactis* by transcriptomic analysis of the lactococcal mevalonate pathway comparing wild-type and recombinant lactococcal strains developed in this study and previous studies.

### Conclusions

The current study specifically describes the use of *L. lactis* as a microbial cell factory for *P. minor* β-sesquiphellandrene. However, on a wider scope, this study represents the use of *L. lactis* as an alternative heterologous host for isoprenoid production. It also describes the first study where the prokaryotic mevalonate pathway has been metabolically engineered for increased isoprenoid production by overexpression of HMGR.
